# Vitamin D and Sport Performance

**DOI:** 10.3390/nu12030841

**Published:** 2020-03-21

**Authors:** Beat Knechtle, Pantelis Theodoros Nikolaidis

**Affiliations:** 1 Institute of Primary Care, University of Zurich, 8091 Zurich, Switzerland; 2 Medbase St. Gallen Am Vadianplatz, 9001 St. Gallen, Switzerland; 3 Exercise Physiology Laboratory, 18450 Nikaia, Greece; pademil@hotmail.com; 4 School of Health and Caring Sciences, University of West Attica, 12243 Athens, Greece

Vitamin D seems to be very important for general health but also for athletic performance [1,2]. Insufficiency in Vitamin D is a serious problem in general internal medicine. Different disorders have been reported to be associated with Vitamin D deficiency. Certain populations such as infants, children, premenopausal women, diverse racial or ethnic groups, and elderly people are at an increased risk for osteoporosis and osteoporotic fractures, among other problems [3,4]. In athletes, certain populations such as women might be at a higher risk for Vitamin D deficiency [5]. Little is known about whether supplementation with Vitamin D in athletes with deficiency in Vitamin D improves performance. One of the purposes of this Special Issue “Vitamin D and Sport Performance“ is to gain more information about the prevalence of Vitamin D deficiency in different sport disciplines and populations. Another purpose is to examine whether the supplementation of certain populations of athletes with Vitamin D deficiency can improve athletic performance in different sports disciplines.

The present special issue on "Vitamin D and Sport Performance" included two reviews [6,7] and six original research articles [8,9,10,11,12,13]. Ksiażek and colleagues [6] reviewed the relevant literature and concluded that Vitamin D deficiency may cause deficits in strength, and lead to degeneration of type II muscle fibers, which has been found to negatively correlate with physical performance. In addition, they highlighted that Vitamin D supplementation has been shown to improve Vitamin D status and can positively affect skeletal muscles. Wiciński and colleagues [7] concluded that the plasma concentration of Vitamin D was associated with muscle function and immune response in both the general and athletic populations.

The original research articles included studies on soccer [8,9,13] and volleyball players [10] focusing on the prevalence of Vitamin D deficiency, the effectiveness of Vitamin D supplementation and the relationship of Vitamin D with performance indices. For instance, Bezuglov and colleagues [9] observed a 92% increase in Vitamin D plasma concentration after a daily supplementation of cholecalciferol for two months in young soccer players. Moreover, Skalska and colleagues [13] reported an increase in 25(OH)D concentration (119%) in a supplemented group and a decrease (8.4%) in a non-supplemented group of soccer players during eight-week high-intensity training. Kim and colleagues [10] found 27% with Vitamin D deficiency, 46% with Vitamin D-insufficient, and 27% of male professional volleyball players with Vitamin D-sufficient, and Vitamin D level did not correlate with shoulder muscle strength. Furthermore, Bezuglov and colleagues [8] observed no difference in 5, 15, and 30 m sprint tests and the standing long jump test between young soccer players with 25(OH)D levels below (serum 25(OH)D <30 ng/mL) or above reference (serum 25(OH)D 61-130 ng/mL). In addition, they showed an increase of 25(OH)D concentration by 79.2% in the group with low 25(OH)D level after a daily supplementation for two months. On the other hand, the original research article of Myśliwec and colleagues [12] concerned boys with type 1 diabetes mellitus, where it was shown that a group with Vitamin D deficiency had likely higher glycemic variability during days of exercise training than those with a suboptimal level of Vitamin D. Finally, Larson-Meyer and colleagues [11] examined the validation of a food frequency and lifestyle questionnaire to assess Vitamin D intake and lifestyle factors affecting status with regard to serum 25(OH)D concentrations and 7-day food diaries. They highlighted the difficulty of utilizing intake methodologies for Vitamin D, as its status is influenced by body size and dietary sources.

It is hoped that this special issue will aid to raise the awareness of the current trends of Vitamin D prevalence and supplementation. Practitioners working with athletes should encourage them to be screened regularly for plasma concentration of Vitamin D level. The articles of this special issue are expected to trigger further research about the relationship of Vitamin D with sport performance.
